# The Analysis of Genetic Aberrations in Children with Inherited Neurometabolic and Neurodevelopmental Disorders

**DOI:** 10.1155/2014/424796

**Published:** 2014-05-13

**Authors:** Krystyna Szymańska, Krzysztof Szczałuba, Agnieszka Ługowska, Ewa Obersztyn, Marek Radkowski, Beata A. Nowakowska, Katarzyna Kuśmierska, Jolanta Tryfon, Urszula Demkow

**Affiliations:** ^1^Department of Clinical and Experimental Neuropathology, Mossakowski Medical Research Centre, Polish Academy of Sciences, 02-106 Warsaw, Poland; ^2^Department of Child Psychiatry, Medical University of Warsaw, 00-576 Warsaw, Poland; ^3^GenCentrum (Regional Center for Clinical Genetics and Modern Technologies), 25-375 Kielce, Poland; ^4^Department of Genetics, Institute of Psychiatry and Neurology, 02-957 Warsaw, Poland; ^5^Department of Medical Genetics, Institute of Mother and Child, 01-211 Warsaw, Poland; ^6^Department of Immunopathology of Infectious Diseases, Medical University of Warsaw, 02-091 Warsaw, Poland; ^7^Clinic of Child and Adolescent Neurology, Institute of Mother and Child, 01-211 Warsaw, Poland; ^8^Department of Laboratory Diagnostics and Clinical Immunology, Medical University of Warsaw, 00-576 Warsaw, Poland

## Abstract

Inherited encephalopathies include a broad spectrum of heterogeneous disorders. To provide a correct diagnosis, an integrated approach including genetic testing is warranted. We report seven patients with difficult to diagnose inborn paediatric encephalopathies. The diagnosis could not be attained only by means of clinical and laboratory investigations and MRI. Additional genetic testing was required. Cytogenetics, PCR based tests, and array-based comparative genome hybridization were performed. In 4 patients with impaired language abilities we found the presence of microduplication in the region 16q23.1 affecting two dose-sensitive genes: *WWOX* (OMIM 605131) and *MAF* (OMIM 177075) (1 case), an interstitial deletion of the 17p11.2 region (2 patients further diagnosed as Smith-Magenis syndrome), and deletion encompassing first three exons of Myocyte Enhancer Factor gene *2MEF2C* (1 case). The two other cases represented progressing dystonia. Characteristic GAG deletion in *DYT1* consistently with the diagnosis of torsion dystonia was confirmed in 1 case. Last enrolled patient presented with clinical picture consistent with Krabbe disease confirmed by finding of two pathogenic variants of *GALC* gene and the absence of mutations in *PSAP*. The integrated diagnostic approach including genetic testing in selected examples of complicated hereditary diseases of the brain is largely discussed in this paper.

## 1. Introduction

Neurometabolic and neurodevelopmental disorders have complex behavioural and cognitive phenotypes and in some cases may cause diagnostic dilemmas. To provide a diagnosis of these challenging medical conditions the patient has to be subjected to an integrated set of clinical, imaging, and laboratory analyses [1–3]. Combining extensive clinical workup with neuroimaging and sophisticated biochemical testing is essential to reach an accurate diagnosis, but some cases still remain unresolved. The analysis of data from broad patient phenotyping, together with genetic testing results, is warranted to attain a diagnosis in difficult cases. Genetic testing provides the final confirmation of clinical suspicions or constitutes an essential addition to prior examinations performed to reach a clinical diagnosis [4, 5]. In the latter case, both normal and abnormal results of such analyses can be of potential value to the clinician. Sometimes, genetic testing may provide completely new, important information about the complex human cognitive functions, including language development. A range of molecular genetic techniques, such as array comparative genomic hybridization, the identification of copy number variations, and in the future sequencing of genes, can support the clinical diagnosis and serve as a rich resource of the knowledge about disease mechanisms [4–6].

The aim of the current study was to analyse the genetic etiologies of congenital disorders of the central nervous system in children.

## 2. Patients and Methods

Seven patients from Polish families with inborn paediatric encephalopathies were retrospectively enrolled. The patients were categorized into 2 subgroups: first group (4 children) with nonprogressive neurodevelopmental disorders and the second group of 3 children with progressive neurological process. The depiction of specific entities only by means of clinical and biochemical analysis was difficult in all patients. Additional genetic testing was required in all cases either to fulfil the criteria of a defined genetic syndrome or to associate known phenotype with a given variant.

All patients underwent detailed examination using an assessment protocol based on a multidisciplinary approach and administered by a child neurologist experienced in developmental disorders. Clinical investigations included family history and medical records of the child's pre- and postnatal period. All children were subjected to an in-depth analysis of the developmental phenotype—motor patterns in terms of gross and fine motor skills and motor functions, social relationships, receptive and expressive language development, behavioural and emotional regulation (mood regulation, attention, sleep/arousal, and feeding behaviour), and cognitive development evaluation. All subjects have undergone detailed neurological examination and have been assessed by psychologists. The clinical characteristics of all the enrolled patients are presented in Table 1.

The developmental and intellectual characteristics of all the enrolled patients are presented in Table 2.

Routine haematology and biochemistry including glucose, ammonia, lactate, and thyroid function tests in plasma were performed. Neuroimaging tests including brain MRI and awake and asleep electroencephalogram were performed in every case. If the patient history and clinical signs, as well as MRI abnormalities, indicated a neurometabolic or neurodegenerative disease, a battery of specific neurometabolic and genetic tests was performed. Depending on the clinical picture, biochemical diagnostics included serum, cerebrospinal fluid and urine amino acids, acylcarnitines analyses in dried blood spot by tandem mass spectrometry (MS/MS), urine organic acids analysis by gas chromatography-mass spectroscopy (GC/MS), lysosomal enzymes activities, urinary oligosaccharides and glycosaminoglycans, plasma ceruloplasmin, biogenic amines metabolites in cerebrospinal fluid (CSF), and pterins in urine. The results of magnetic resonance imaging (MRI), electroencephalography (EEG), and metabolic testing are shown in Table 3.

Genomic DNA was extracted from patients' fresh blood samples. Molecular cytogenetic, PCR, and/or array-based comparative genome hybridization were performed in examined individuals. Array CGH was performed using 180 K microarray: 4 × 180 K from Agilent (Agilent 180 K, 021924). DNA digestion, labeling, and hybridization were performed according to the manufacturer's instructions. Scanned images were quantified using Agilent Feature Extraction software (v10.0). The customized web2py software was used for genomic copy-number analysis. All genomic coordinates are based on the NCBI36/hg18 reference genome.

For DYT1 mutation, DNA was extracted from whole blood following standard protocols. We used published primers, 6418 and 6419 [7], for PCR amplification across the critical region of the DYT1 gene. PCR products were resolved in a denaturing 6% polyacrylamide gel and visualized by silver staining.

Karyotype analysis was performed using peripheral blood lymphocyte cultures and a standard GTG banding method [8].

FISH analysis was performed in phytohemagglutinin-stimulated peripheral blood lymphocytes using standard procedures with LSI SMS (Vysis) probes specific for 17p11.2 Smith-Magenis syndrome critical region.

GCH1, GALC, and PSAP sequencing and electrophoresis experiments were done according to standard protocols and manufacturer's instructions on ABI Prism Genetic Analyzer 310 (Applied Biosystems).

The results of genetic analysis are presented in Table 4.

## 3. Discussion

Genetic testing is the optimal strategy in patients with complex, multisystem disorders in whom a disease cannot be identified despite exhaustive diagnostic efforts. Inherited alterations driving neurodevelopmental disorders are complex, including not only de novo mutations and common, low-risk polymorphisms but also high or moderate risk variants including copy number variation. In the current study we report 7 difficult clinical cases of congenital disorders of the brain diagnosed on the basis of integrative approach including extensive clinical and radiological examination, biochemical analysis, and genetic testing. Enrolled patient belonged to the two groups. First group included children with intellectual disability and the other group consisted of children with neurometabolic disorders. The clinical symptoms of those conditions most frequently appeared in the first years of life. Depending on the affected structure or function, various clinical pictures were present, being the starting point for further diagnostics. Basically routine clinical and laboratory investigations, MRI imaging of the central nervous system, and specialized metabolic testing did not yield a correct diagnosis.

Four patients (numbers 1–4) displayed speech and language difficulties of varying degrees. In patient number 1 both expressive and receptive language abilities were impaired to a greater extent than could be expected on the basis of child IQ. Interestingly, no pragmatic disorder has been observed and child successfully communicated using gestures and onomatopoeia. Array CGH revealed presence of microduplication in the region 16q23.1 with the size ranging from 0.744 Mb to 0.827 Mb. The variant has never been described in the literature before. Its parental origin could not be fully traced either; however, copy-number variations in the 16q22-q24 region have been linked to autism and developmental delay phenotypes [9, 10]. The duplication found in Patient 1 affects two dose-sensitive genes:* WWOX* (OMIM 605131) and* MAF* (OMIM 177075). Human WW domain-containing oxidoreductase (WWOX, FOR, or WOX1) induces apoptosis, probably via the mitochondrial pathway and is a proapoptotic protein and a tumour suppressor [11]. In rats, it was found that WOX1 plays an essential role in the 1-methyl-4-phenyl-pyridinium induced neuronal death and is present in the condensed nuclei and damaged mitochondria of degenerative neurons in the striatum and cortex, ipsilaterally to intoxication [12]. Protein encoded by the* MAF* gene (v-maf avian musculoaponeurotic fibrosarcoma oncogene homolog (c-MAF)) is a DNA-binding leucine zipper-containing transcription factor [13, 14]. The expression of transcription factor c-Maf plays an important role in the development of interneurons of laminae III/IV in the dorsal horn of mouse spinal cord, which receive inputs from mechanoreceptive dorsal root ganglion neurons [14].

C-MAF-inducing protein (CMIP) is involved in the c-maf signalling pathway. CMIP interacts with filamin A (plays an important role in the formation of the dendritic spine) and the NF-kappaB subunit RelA (in a mouse model it plays a role in memory formation, cognition, and behaviour; NF-*κ*B signalling pathway is altered in many chronic neurodegenerative diseases in humans). CMIP regulates nonword repetition performance and modulates phonological short-term memory commonly impaired in specific language impairment (SLI) [15].* CMIP* was recently recognized as one of the important genes involved in the aetiology of specific language impairment [16]. It is difficult to attribute deep speech and language impairment, which dominates in the clinical picture of this patient, to the duplication of a specific gene, but one should consider the eventuality of the interaction of the c-maf signalling pathway and CMIP. In the course of clinical observation these patients decelerated overall mental development with age (especially deductive reasoning, generalization, anticipation, planning, and construction abilities). Initially, development dynamics were constant but then slowed down or maybe even deteriorated. This could be due to the dysfunction of WWOX, that is, its impact on apoptosis. The significance of this change in our patient is limited by the lack of paternal DNA and detailed clinical data about speech development in the father.

The language problems of patient number 2 were further blunted by impaired social development and joint attention. Speech comprehension in this child was relatively good. In patient number 3 speech difficulties were secondary to hearing impairment. Following the implantation of a cochlear implant the patient quickly improved verbal communication skills. In both patients genetic testing allowed to diagnose the Smith-Magenis syndrome (SMS), usually a sporadic disorder characterized by dysmorphic features, hypotonia, developmental delay, speech difficulties, sleep disturbance, and behavioural problems (hyperactivity, aggression, and autoagression) [17]. In both cases a deletion affecting the same genes: the human homologue of the Drosophila flightless-I gene (*FLII*); cytosolic serine hydroxymethyltransferase (*SHMT1*); 21,23 the human homologue of Drosophila lethal 2 giant larva (*LLGL1*); and 18 topoisomerase IIIa (*TOP3A*), was found confirming the clinical diagnosis of SMS. Nevertheless, the phenotypic differences between both patients cannot be explained only on the basis of the performed genetic tests.

Patient number 4 previously described by Nowakowska et al. [18] suffered from very deep speech impairment. The lack of speech and language development was accompanied by deep mental retardation, autistic symptoms, and tremor. Moreover, this patient displayed an atypical movement pattern called mirror movement of the upper limbs, which persisted even in his second/third year of life. Mirror movements (m. m.) are involuntary movements executed by one side of the body during voluntary movements of the contralateral homologous body parts. This is a physiological phenomenon, which normally occurs at the early stage of development [19]. It is clearly visible in the first months of life, but in children older than 6 months it is no longer the dominant pattern and completely disappears by the 10th month. The persistence of this symptom up to 3–5 years can be explained by delayed/abnormal maturation of inhibitory processes in the brain. The overall movement decomposition and wide-based stance and gait were also noted pointing to developmental cerebellar dysfunction. Furthermore, an impact of retarded myelination on the boy's clinical picture cannot be excluded. Genetic testing of this patient revealed a deletion encompassing the first three exons of* MEF2C*. MEF-2 (Myocyte Enhancer Factor 2) proteins are transcription factors that belong to the MADS (MCM1, Agamous, Deficiens, and serum-response factor) box family of transcription factors. In mammals there are four isoforms MEF-2 A, B, C, and D encoded in four genes; the expression of which overlaps in developing muscle and neural cells during embryogenesis [20]. MEF2C is expressed preferentially in certain neuronal layers of the cortex and that expression declines during postnatal development [21]. MEF2C plays a crucial role in the homeostatic control of activity-dependent synaptogenesis [12]. It is an important process in the establishment of functional neuronal circuits during development and memory storage [22]. Barbosa and coworkers proved that the deletion of the MEF2C transcription factor in mouse brain impairs hippocampal-dependent learning and memory [22]. MEF2C plays an essential role in neurodevelopment by suppressing the number of excitatory synapses during activity-dependent refinement of synaptic connectivity and thus regulating basal and evoked synaptic transmission without affecting synapse structure [22]. A strong dominance of excitation over inhibition observed in the described patient could be related to this mechanism.

It is generally recognized that speech and language disorders tend to cluster in families; therefore, a genetic testing is always warranted. The first description of a family with severe speech and language impairments dates back to 1990 [23]. It was related to a mutation within the* FOXP2*, the first known gene associated with communication disorders.* FOXP2* is a member of the forkhead family of transcription factor genes and plays a key role in brain development [24]. A systematic genetic analysis of developmental speech disorders could contribute to defining specific phenotypes of language impairments and facilitate the diagnosis and treatment of such conditions. Genetic studies in this group of children may reveal molecular etiology of speech impairment in a single patient; moreover, the identification of genes linked to language phenotypes and further characterization of normal and aberrant functions of these genes can provide valuable insight into the biological foundations of complex symbolic form of communication that speech and language represent. On the other hand, genetic testing of a subset of genes associated with speech and language development can be screened in children with speech and language disorders in order to guide early and effective therapy.

The other examined group consisted of three patients with neurometabolic and movement disorders presenting as various neurological syndromes. The detailed analysis of those patients clearly shows that it is not possible to reach a correct diagnosis of neurometabolic or neurodegenerative disorder in children only on the basis of clinical features, neuroimaging, and electrophysiological examinations. Both detailed biochemical analyses of different body fluids, including CSF, accompanied with genetic testing are crucial to recognize a specific disease entity and to implement appropriate therapy.

Patient number 5 presented with a fast progression of dystonia beginning from the trunk muscles while cognitive functions remaining unimpaired. The normal results of urine organic acid analysis by GC/MS and a normal brain MRI allowed excluding organic acidurias (e.g., glutaric aciduria type I) and pantothenate kinase-associated neurodegeneration. The diagnosis of autosomal dominant GTP-cyclohydrolase I (GTPCH I) deficiency was excluded as the early impairment of trunk muscles and a serious deterioration following L-dopa treatment is unusual for those disorders. In this case, PCR-RFLP analysis revealed the presence of characteristic GAG deletion in the* DYT1* consistently with the diagnosis of torsion dystonia. The analysis of this case clearly shows that analysis of* DYT1* is necessary to confirm or exclude the diagnosis of torsion dystonia.

The clinical picture of patient number 6 pointed to l-dopa responsive dystonia without hyperphenylalaninemia, which may occur in autosomal dominant GTPCH I deficiency, sepiapterin reductase (SR) deficiency, or in tyrosine hydroxylase (TH) deficiency. The diagnosis of inherited biogenic amine metabolism disorders (BAD) is almost exclusively based on the quantitative determination of the metabolites in CSF [25].

The characteristic clinical symptoms like diurnal variation and dopa-responsive dystonia, analysis of biogenic amine metabolites in CSF, pterin profile in CSF/urine, and phenylalanine loading test are usually sufficient for diagnosis. Genetic tests are primarily required to distinguish between the two forms of GTPCH (autosomal dominant versus autosomal recessive form). This information is crucial for genetic counselling of the family.

One limitation of AD GTPCH I deficiency testing is that molecular analyses in the coding region cannot identify the mutations in this gene in approximately 40% of patients [26]. In some patients a large gene deletion or an intragenic duplication or inversion of the* GCH1* gene or abnormalities in noncoding regions of the gene can cause an enzyme deficit. Such cases require the use of other methods, such in next generation sequencing order to recognize intragenic copy-number variation. As in patient number 6 molecular investigation did not detect a pathogenic mutation in the* GCH1* gene and genetic examination; without a prior analysis of biogenic amine and pterin metabolites in CSF, it would have failed to identify the cause of the defect.

In patient number 7 [27] clinical picture was consistent with Krabbe disease and borderline beta-galactocerebrosidase activity pointed to the impairment in a pathway of galactocerebroside degradation. The high residual activity of beta-galactocerebrosidase was not typical of infantile-onset Krabbe disease and suggested the deficiency of saposin A [28]. The clinical picture and prognosis in both defects does not differ much. The sequencing of the* GALC* gene revealed two pathogenic variants and the absence of mutations in the* PSAP* gene, thus confirming the diagnosis of globoid leukodystrophy [27]. Proper molecular diagnostics has enabled genetic counselling and family planning for this family.

## 4. Closing Remarks

The vast majority of neurometabolic and neurodegenerative diseases belongs to the group of rare diseases (frequency less than 1 : 2000 live births). These sporadic diseases are diagnosed with delay. The availability of new modalities of genetic testing like microarray high-resolution CGH have greatly contributed to the assessment of copy number variation and their role in diverse phenotypes. Moreover, whole genome association studies and exon sequencing in orphan diseases have revealed new candidate genes. In this work, we have taken advantage of array CGH to analyze genomic DNA in patients with the aim to identify potential molecular variants that could be associated with differential clinical outcomes. The proper paediatric and neurological examination together with neuroimaging and biochemistry supplemented with genetic testing was necessary to confirm or rule out the diagnosis. This study also highlights the advantage of an integrated approach to a patient with complicated hereditary disease to establish a proper and prompt diagnosis.

## Figures and Tables

**Table 1:** The clinical characteristics of enrolled subjects.

Patient number, sex/age, family history (FH)	Course of pregnancy, perinatal period (PP)	First symptoms	Age at the time of diagnosis	Dysmorphic features	Clinical symptoms
Patient 1, female 18 yrs, FH-negative	Premature uterine contractions, PP-uneventful	2 yr develop mental delay	16 yr	yes	Dysmorphic features; convergent strabismus of the left eye, hirsutism; scoliosis; epilepsy—valproic acid resulted in a complete EEG normalization; simple stereotypic movements of the upper limbs

Patient 2, male 7 yrs, FH-negative	Premature uterine contractions, PP-uneventful	1 mo dysmorphic features	1.8 yr	yes	Dysmorphic features; neonatal oedema of the legs, cryptorchidism, brachydactyly; short stature; pronounced hypotonia; gastroesophageal reflux; severe sleep disturbances; severe autoagression, aggression, inadequate long temper tantrums, and stereotypic movements

Patient 3, female 6 yrs, FH-negative	Gestational diabetes, PP-uneventful	1 mo dysmorphic features	2 yr	yes	Dysmorphic features; poor suck, hypoacusia-cochlear implant, significant hypotonia, no sleep disturbances, cheerful, without aggression or autoagression

Patient 4, male 7.7 yrs, FH-negative	Mother's hyperthyroidism and toxoplasmosis, birth at 35 w., 2480 g 10 p. Apgar	2 mo abnormal movements	2.10 yr	yes	Dysmorphic features, vertical and horizontal nystagmus up to 5 mo, global hypotonia, ataxia, decomposition of the movement. Obligatory mirror movement of upper limbs up to 3–5 yrs, exaggerated startle for unexpected stimuli with head retraction and trunk retropulsion and tremor of stiff limbs; decreased pain sensation. Epilepsy improvement after lamotrigine therapy.

Patient 5, male 21 yrs, FH-negative	No foetal or perinatal problems	5 yr dystonia	8 yr	no	Motor deterioration, dystonia; 8 yr—only slight voluntary movements of the left upper limb, forced posture, increased muscle tone (rigidity), deep tendon reflexes symmetrical, bilateral dorsal hallux sign; treatment with l-dopa led to deterioration; improvement after bilateral deep brain stimulation of the internal pallidum

Patient 6, female 26 yrs, FH-negative	No foetal or perinatal problems	4 yr dystonia	13 yr	no	Since 4-5 years of age she began falling and her gait was compromised. Dystonia; remission after l-dopa therapy

Patient 7, male, died at 15 mo, FH-negative	No foetal or perinatal problems	Before 6 mo tetraparesis	13 mo	no	Motor deterioration; tetraparesis spastica

**Table 2:** The developmental and intellectual characteristics of enrolled patients.

Patient number	Development of gross motor skills	Development of fine motor skills	Speech and language development	Intellectual functioning	Autistic features
Patient 1	Abnormal motor coordination	Significant deficits in praxis and visual-motor coordination	Speech, especially active, not developed (single words or phrases, often the first syllable instead of the full word). Oral dyspraxia.	6 yr—nonverbal Leiter scale IQ-75; 11 yr and 13 yr—nonverbal scale of the WISC-R test IQ 52; 17 yr—nonverbal scale of the WISC-R test IQ 42	no

Patient 2	Gross motor function-clumsy, probably partly due to significant hypotonia	Fine motor skills-retarded: at age of 3 developed the pincer grasp	Active and passive speech delayed. At age of 3—a few sounds (not naming objects, not imitating any sounds or words), communication using gestures.	Mental retardation with autistic features; symbolic play absent.	yes

Patient 3	Walked alone at the age of 26 mo.	Abnormal	Deficits of speech connected with hypoacusia and defective articulation	Cognitive development at 2 yr corresponding with the chronological age; symbolic play was present. 5 yr—nonverbal Leiter scale IQ-99	no

Patient 4	Began to walk alone in the third year of life-shaky, wide-based stance and gait	“Jerky movements” with reduced complexity, variability, and fluency. Up to 5 yr of age no pincer grasp.	7 yr—total lack of spoken language, reacted to very simple commands.	ASD with profound mental retardation	Profound

Patient 5	Dystonia at 5 yr	Dystonia at 5 yr	Normal	Cognitive functions normal	no

Patient 6	Dystonia at 4 yr	Dystonia at 4 yr	Normal	Cognitive functions normal	no

Patient 7	Abnormal due to tetraparesis spastica	Abnormal due to tetraparesis spastica	Lack of development	Lack of development	no

**Table 3:** The results of MRI imaging, EEG, and metabolic testing in all enrolled subjects.

Patient number	Brain ultrasound	Brain MRI	EEG	Biochemical diagnostics tests abnormalities
Patient 1	Not performed	At the age of 5 yr T2-hyperintense changes of the periventricular white matter around the centre of the body of the lateral ventricles. 18 yr—normal	Generalized spikes and spike-and-wave discharges (2.5–5 Hz) as well as focal spikes	Not found

Patient 2	12 mo.—dilatation of the lateral ventricles (11 and 14 mm)	Not performed	Not performed	Hypogammaglobulinemia; mild elevation of alpha-fetoprotein (13 IU, reference value up to 5.5 IU).

Patient 3	Dilatation of the lateral ventricles	Dilatation of the lateral ventricles	Normal	Not found

Patient 4	Normal	10 mo abnormal-mild thinning of the corpus callosum and delayed white matter myelination	Ictal activity in the form of slow waves with sharp waves and spikes	Strong signal of 2-ketoglutaric acid in urine; elevation of alpha-fetoprotein (13 IU/normal up to 5.5 IU/).

Patient 5	Not performed	Normal	Normal	Not found

Patient 6	Not performed	Normal	Normal	Decreased concentrations of HVA and 5-HIAA in CSF. Pterine profile in urine—very low concentration of neopterin and biopterin, below the reference range: 0.16 mmol/mol creat (ref. range: 0.3–4.0) and 0.27 mmol/mol creat (ref. range: 0.5–3.0), respectively. Phenylalanine loading test in blood—Phe concentration and Phe to Tyr ratio significantly increased after 1 h, 2h, and 4 h.

Patient 7	Not performed	T2-hyperintense changes of the periventricular white matter and cerebellar white matter	Not performed	*β*-galactocerebrosidase—in two different samples of blood leukocytes revealed 2.1 and 2.0 nmol/mg protein/18 hr, respectively (reference values: 4–9.4 nmol/mg protein/18 hr; in patients with Krabbe disease below 2.0 nmol/mg protein/18 hr). In cultured skin fibroblasts: 3.7 nmol/mg protein/18 hr (reference values 3.9–15.2 nmol/mg protein/18 hr).

HVA: homovanillic acid (dopamine metabolite); 5-HIAA: 5-hydroxyindoleacetic acid (a serotonin metabolite); Phe: phenylalanine; Tyr: tyrosine.

**Table 4:** Results of genetic testing in all enrolled patients. All genomic coordinates are based on the NCBI36/hg18 reference genome.

Patient number	Cytogenetic/molecular tests	Detected variant (start–end hg18)	Size (Mb)	Inheritance
1	Array CGH	arr 16q23.1 (77,445,915–78,190,209) dup	0.744–0.827	Unknown

2	GTG karyotype	del(17)(p11.2)	—	De novo

3	Fluorescence in situ hybridization	ish del(17) (p11.2p11.2)	—	De novo

4	Array CGH	arr 5q14.3 (88,121,748–88,232,276) del	0.111–0.148	De novo

5	*DYT1* PCR	c.907_909delGAG	—	De novo

6	*GCH1* sequencing	Not found	—	—

7	*GALC* and *PSAP* sequencing	30 kb deletion encompassing exons 11–17 within the *GALC* gene; mutation in exon 11 (c.1186C>T, p.R396W)	—	—
